# Mangiferin Induces Post-Implant Osteointegration in Male Diabetic Rats

**DOI:** 10.3390/medicina60081224

**Published:** 2024-07-28

**Authors:** Bünyamin Ongan, Ömer Ekici, Gökhan Sadi, Esra Aslan, Mehmet Bilgehan Pektaş

**Affiliations:** 1 Department of Oral and Maxillofacial Surgery, Faculty of Dentistry, Afyonkarahisar Health Sciences University, 03200 Afyonkarahisar, Türkiye; b.ongan.1907@gmail.com (B.O.); omer.ekici@afsu.edu.tr (Ö.E.); 2 Department of Biology, K.O. Science Faculty, Karamanoglu Mehmetbey University, 70100 Karaman, Türkiye; sadigokhan@gmail.com; 3 Department of Histology and Embryology, Faculty of Medicine, Afyonkarahisar Health Sciences University, 03200 Afyonkarahisar, Türkiye; dr_esragul@hotmail.com; 4 Department of Medical Pharmacology, Faculty of Medicine, Afyonkarahisar Health Sciences University, 03200 Afyonkarahisar, Türkiye

**Keywords:** diabetes, mangiferin, osteointegration, bone repairing, dental implant

## Abstract

*Background and Objectives:* Hyperglycemia is known to undermine the osteointegration process of implants. In this study, the effects of mangiferin (MF) on the post-implant osteointegration process in a type-II diabetes model were investigated molecularly and morphologically. *Materials and Methods:* Sprague Dawley male rats were divided into three groups: control, diabetes, and diabetes + MF. All animals were implanted in their tibia bones on day 0. At the end of the 3-month experimental period, the animals’ blood and the implant area were isolated. Biochemical measurements were performed on blood samples and micro-CT, qRT-PCR, histological, and immunohistochemical measurements were performed on tibia samples. *Results:* MF significantly improved the increased glucose, triglyceride-VLDL levels, and liver enzymes due to diabetes. By administering MF to diabetic rats, the osteointegration percentage and bone volume increased while porosity decreased. DKK1 and BMP-2 mRNA expressions and OPN, OCN, and OSN mRNA–protein expressions increased by MF administration in diabetic rats. Additionally, while osteoblast and osteoid surface areas increased with MF, osteoclast and eroded surface areas decreased. *Conclusions:* The findings of our study indicate that MF will be beneficial to the bone-repairing process and osteointegration, which are impaired by type-II diabetes.

## 1. Introduction

Diabetes is a metabolic disorder characterized by hyperglycemia, which can result in chronic inflammation and tissue damage. Chronic hyperglycemia causes complications in various organs such as eyes, kidneys, nerves, heart, and blood vessels [1,2,3,4]. Hyperglycemia also leads to various oral health problems especially periodontal diseases [5]. Periodontitis is considered the 6th most common complication of diabetes. There is a bidirectional relationship between type-II diabetes and periodontitis so the risk of periodontitis is increased in the case of type-II diabetes [6]. An experimental model of type-I diabetes accompanied with periodontitis in the upper second molars of rats showed that diabetes-induced gingival tumor necrosis factor-α (TNFα) and inducible nitric oxide synthases (iNOSs) aggravated inflammation and triggered alveolar bone resorption [7].

Bone healing is frequently impaired in diabetic patients, and it is usually considered that this impairment is related enhanced apoptosis of osteogenic cells and their progenitors by hyperglycemia [8]. Bone marrow stromal cells (BMSCs) are one of the key progenitors of osteogenic cells in the periodontium. Hyperglycemia harms their activity and accelerates their apoptosis which can suppress alveolar bone formation [9].

It has been shown that diabetes prolongs the healing of bone tissue within the calvarial defects in a rat model of diabetes [10]. Another experimental model of hyperglycemia demonstrated that a diet rich in corn syrup damaged the craniomandibular bone and suppressed bone development in rats [11]. The same model also indicated that hyperglycemia causes inflammation in the masseter and gingival tissue, and it was stated that this situation may cause prolongation in the healing of soft tissues [12]. It is known that diabetes and/or hyperglycemia exert negative effects on osteointegration in patients undergoing dental implant restoration [13]. Prolonged hyperglycemia may ultimately lead to secondary complications associated with diabetes, such as microvascular disease, delayed wound healing, and susceptibility to infection, which may interfere with the osteointegration of dental implants [14,15]. 

Diabetes and diabetes-related complications are health problems that do not have radical treatment. There are many herbal medicines which have attracted much attention as potential therapeutic agents in the prevention and treatment of diabetic complications [16]. Mangiferin (MF) (1,3,6,7-tetrahydroxyxanthone-C2-β-Dglucoside) is a bioactive ingredient predominantly isolated from the mango tree, with potent antioxidant activity and pharmacological potential for antidiabetic, antitumor, lipometabolism regulating, cardioprotective, antihyperuricemic, neuroprotective, antioxidant, anti-inflammatory, antipyretic, analgesic, antibacterial, antiviral, and immunomodulatory effects [17]. Its bioactivity can be considered as non-toxic as its reported oral LD_50_ value in mice was 400 mg/kg, so it has become a promising candidate for developing natural remedies for obesity, diabetes, and cardiovascular disease [17,18]. 

MF inhibits hyperglycemia by regulating inflammatory molecules like protein kinase C (PKC), peroxisome proliferator-activated receptor c (PPARc), and Nf-κB [19]. Moreover, MF regulates various transcription factors like nuclear factor kappa B (Nf-κB) and nuclear factor erythroid 2-related factor-2 (Nrf-2) and modulates the expression of different proinflammatory signaling intermediates like TNFα and COX-2 which contribute to its anti-inflammatory and antidiabetic effects [19]. In a model of streptozotocin-induced diabetes, blood glucose, glycosylated hemoglobin, aspartate aminotransferase (AST), alanine aminotransferase (ALT), and alkaline phosphatase (ALP) levels were significantly reduced in MF-treated mice [20].

Osteoblast adhesion is the initial step in the early osteointegration of implants. This step consists of cell migration, proliferation, and differentiation. Persistent hyperglycemia results in the generation of reactive oxygen species (ROS) by increasing mitochondrial oxygen consumption, disrupting mitochondrial function, and activating nicotinamide adenine dinucleotide phosphate (NADPH) oxidase [21]. ROS cause oxidative damage in osteoblasts by inhibiting the expression of antioxidant enzymes Nrf-2 and hemoxygenase-1 [22] and increasing osteoblast apoptosis through the inhibition of the phosphoinositide-3-kinase (PI3K)/protein kinase B (AKT) signaling pathway [23]. Thus, hyperglycemia-induced ROS inhibit the osteogenic capacity of osteoblasts and increase bone resorption by promoting osteoclast differentiation [24]. MF has high antioxidant activity and acts as a scavenger for ROS. It has been reported that MF hinders inflammation by inducing the Nrf-2 pathway [25,26].

MF might have improving effects on bone damage and inflammation in the case of peri-implantitis [27]. In addition, MF blocks TNF-induced Nf-κB activation which could result in the regression of osteolytic bone diseases [28]. An in vitro modeling of periodontal disease points out that MF alleviates inflammatory response by inhibiting the expression of interleukin-6 (IL-6) and activation of Toll-like receptor (TLR) signaling [29]. MF may ameliorate alveolar bone disease through its protective effects on BMSCs [30]. That is, MF inhibits osteoclast formation and bone resorption by attenuating receptor activator of nuclear factor kappa Β ligand (RANKL)-induced signaling [31]. These reports suggest the potential therapeutic effect of MF on the bone remodeling process. The present study aims to specify whether MF administration would have beneficial effects on the biochemical and metabolic parameters, mRNA expressions of bone markers, immunohistochemistry, and histochemical staining of bone tissue in a rat model of diabetes mellitus. 

## 2. Materials and Methods

### 2.1. Animals

The study protocol was approved by the Institutional and Ethical Committee of Animal Research at Afyon Kocatepe University (49533702/142). All experimental procedures were performed according to the Turkish Regulations on the Protection of Animals Used for Experimental and Other Scientific Purposes, the Breeding Places of Experimental Animals, the Establishment, Operation, Inspection, Procedures and Principles of the Laboratories that Undertake Experiments which were updated in 2010 [32].

A total of 24 male *Sprague Dawley* rats which were aged three months and weighed 300–330 g were obtained from Afyon Kocatepe University Experimental Animal Application and Research Center. A power analysis was carried out to determine that a sample size of 24 rats which were homogeneously allocated into three groups (controls, diabetic rats, and diabetic rats receiving MF) had 87.6% power to detect a difference at the 0.05 significance level. Standard rodent chow (Korkutelim Yem Sanayi A.Ş., Antalya, Türkiye) that was composed of 62% starch, 23% protein, 4% fat, 7% cellulose, vitamins, and salt mixture was given ad libitum with drinking water during the experiment. All rats were acclimatized for one week prior to experimentation and then three groups were formed for control, diabetes, and diabetes + MF.

### 2.2. Implant Placement

To investigate osteointegration, specially produced dental implants with hexagonal platforms were surgically placed on the tibia in all rats. These specially produced dental implants had a diameter of 2.5 mm, length of 4 mm, and resorbable blast media (Implance Implant Systems, AGS Medical, Istanbul, Türkiye). 

**Figure 1:**
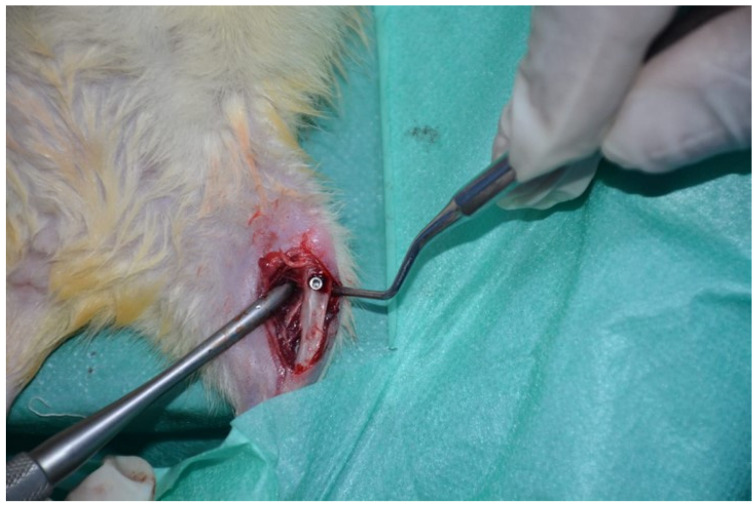
Implant placement at proximal tibia of the rat.

To perform surgery for implant placement, all animals were anesthetized with 10–20 mg/kg xylazine (Rompun 2%, Bayer, Istanbul, Türkiye) and 50 mg/kg ketamine HCl (Ketalar, Eczacıbaşı-Warner Lambert, Istanbul, Türkiye). Then, the skin corresponding to the proximal part of tibia was shaved bilaterally and an antiseptic solution was applied. Following intramuscular administration of prophylactic antibiotics (50 mg/kg ceftriaxone, Cephaxon, Toprak Drug Co., İstanbul, Türkiye) and analgesics (4 mg/kg carprofen, Rimadyl, Pfizer, New York, NY, USA), the tibia was fixed to the operating table and the surgical area was covered with a sterile drape. Afterwards, an incision was made in the skin above the tibia by using a number 15 scalpel and the metaphyseal part of the bone was reached (Figure 1). While opening the slots for the placement of implant, the surgical area was continuously washed with sterile saline to prevent excessive heat. After the implants were placed, the soft tissues were sutured with 4-0 absorbable suture (polyglactin). All rats received 50 mg/kg ceftriaxone intramuscularly and 4 mg/kg carprofen subcutaneously during the three days following surgery. 

### 2.3. Study Protocol

After the placement of tibial implants, all of the rats were allowed to recover uneventfully from anesthesia. The rats that were randomly allocated into the diabetes group and another group of 8 rats that were randomly assigned into the diabetes + MF group received an injection of sterilized and freshly prepared streptozotocin (35 mg/kg/i.p.; Sigma Chemical Co., St. Louis, MO, USA 18883-66-4) in normal saline. After the injection of streptozotocin in saline, the rats were monitored for serum glucose concentrations for 72 h and it was made sure that serum glucose levels exceeded 200 mg/dL for all rats included in the diabetes and diabetes + MF groups. Serum glucose levels in blood were measured by a glucometer (Accucheck Performa, Roche Diagnostics, Mannheim, Germany). The present study aims to specify whether MF administration would have beneficial effects on the biochemical parameters, mRNA expressions of bone markers, immunohistochemistry, and histochemical staining of bone tissue in a rat model of diabetes mellitus. At the same time, the remaining 8 rats which formed the control group had an injection of normal saline at the same volume.

Eight rats in the control group and eight rats in the diabetes group were fed with 1 mL of corn oil (M2886-Cymit Química, S.L. Barcelona, Spain) by gavage whereas eight rats in the diabetes + MF group were fed with MF (40 mg/kg) dissolved in 1 mL corn oil by gavage for 90 days. After the completion of the experiment, all animals were decapitated under anesthesia provided by a combination of ketamine (100 mg/kg) and xylazine (10 mg/kg). Bone–implant complex and the tibia bone samples from the implant site were isolated and stored in 10% formalin solution at −85 °C for genetic, radiological, histological, and immunohistochemical examination. Cardiac blood samples were collected and centrifuged at 4 °C and 10,000× *g* for 10 min.

### 2.4. Biochemical Measurements

The serum separated after centrifugation was taken with a Pasteur pipette, put in *Eppendorf* tubes, and frozen at −85 °C. Triglyceride, VLDL, creatinine, urea, uric acid, ALT, AST, ALP, phosphorus, and sodium (Na) levels in serum samples were measured by a Roche Cobas C501 autoanalyzer using Roche brand commercial kits (Roche Diagnostics International Ltd., Rotkreuz, Switzerland).

### 2.5. Gene Expressions with Real-Time Polymerase Chain Reaction

Total RNAs were isolated from 50 mg of tibial tissues with a RNeasy total RNA isolation kit (Qiagen, Venlo, the Netherlands). After isolation, the amount and quality of total RNA were determined by spectrophotometry at 260/280 nm with a ThermoScanGO microplate reader (Thermo Fisher Scientific, Waltham, MA, USA). Any RNA degradation was detected with agarose gel electrophoresis. cDNA synthesis was carried out by using 500 ng of total RNA and oligo(dT)18 primer using a commercial first strand cDNA synthesis kit (MBI Fermentas, Vilnius, Lithuania). Gene expressions of proteins of interest were identified with real-time PCR by mixing 1 μL cDNA and 5 μL SYBR Green Mastermix (Roche FastStart Universal, SYBR Green Master Mix, Mannheim, Germany). Then, primer pairs at 0.5 mM final concentrations were added to a final volume of 10 μL (Table 1). The real-time program of the quantitative PCR (LightCycler480 II, Roche, Basel, Switzerland) was arranged as follows: initial denaturation at 95 °C for 10 min, denaturation at 95 °C for 10 s, annealing at 58 °C for 15 s, and extension at 72 °C for 15 s with 45 repeated thermal cycles measuring the green fluorescence at the end of each extension step. PCR reactions were performed in triplicate and specificity of PCR products was checked by melt analysis. Negative controls lacking template were additionally used in all reactions. Relative expressions of genes with respect to internal control GAPDH were calculated with the efficiency corrected advance relative quantification tool of the LightCycler^®^ 480 SW 1.5.1 software.

### 2.6. Imaging of the Bone–Implant Complex

Radiological analysis was conducted in radiology laboratories of the Dentistry Faculty at Ankara University. The tibia–implant samples were scanned on a microcomputed tomography (CT) scanner (Skyscan 1275, Skyscan, Kontich, Belgium) with a pixel size of 10 μm. The X-ray tube voltage was 100 kV and the current level was 100 μA. Exposure time was 49 ms. X-ray projections were acquired at 0.20° intervals with a scan angular rotation of 360°. Subsequent reconstructions of the raw data obtained during this scanning phase were acquired using the software program NRecon version 1.7.4.6 (NRecon, Skyscan, Kontich, Belgium). The 8-bit gray images reconstructed by NRecon were imported into the CTan software program (version 1.19.11.1, Skyscan, Kontich, Belgium). Baseline values such as bone volume, bone–implant connectivity density, connectivity, osteointegration percentage, porosity, and total pore volume were recorded at selected regions of interest (ROIs).

### 2.7. Immunohistochemistry

Immunohistochemical staining was carried out by the streptavidin–biotin–peroxidase method. Thus, 5 µm sections were taken from paraffin blocks and these sections were deparaffinized in xylene for 45 min. After being passed through a descending series of ethyl alcohol (100–70%), these sections were transferred to distilled water. To reveal the antigen, conditioning (citrate buffer, pH = 6) was applied in a microwave oven at a medium setting for 20 min. The sections were cooled to room temperature and then washed with PBS 3 times for 2 min. To mask endogenous peroxidase, the sections were treated with 3% H_2_O_2_–methanol for 13 min and afterwards washed with PBS 3 times for 2 min. To avoid unwanted antibody binding, the tissue was covered by 100 µL of blocking solution for 10 min. Primary antibodies OPN (sc-21742, 1/100), RANKL (sc-52950, 1/100), OCN (sc-365797, 1/100), OSN (sc-73472, 1/100), and decorin (DCN) (sc-73896, 1/100) were used overnight in a humidified environment at +4 °C in the refrigerator. All primary antibodies were obtained from Santa Cruz Biotechnology (Dallas, TX, USA). The next day, the sections were washed with PBS 3 times for 2 min and treated with secondary antibody (UltraVision Detection System Anti-Polyvalent HRP Secondary Kit, Thermo-TP-125-HL, Thermo Fisher Scientific). Later, the sections were treated with biotin-labeled secondary antibody (Biotinylated Goat Anti-Polyvalent, Darmstadt, Germany) for 15 min. After being washed with PBS 3 times for 2 min and treated with streptavidin–peroxidase for 15 min, the sections were washed with PBS 3 times for 2 min. The sections were incubated with substrate–chromogen (AEC = 3, aminoethyl 9, carbosol) solution for 10 min until a color reaction (red) was observed. After the sections were put into distilled water, they were counterstained with *Mayer’s* hematoxylin for one minute and rinsed in tap water. Covering agent (Aquose mounting-Lab Vision, Darmstadt, Germany) was dropped and enveloped with a slip. 

For immunochemistry, at least 100 cells were marked in randomly selected areas under ×200 magnification. Scoring was performed by a semiquantitative method in which the percentage of cells stained in each section and the degree of staining were specified. Percentages of positively stained cells were ascertained as 0 (no staining), +1 (marked staining), +2 (prominent staining), +3 (intense staining). Statistical analysis was performed after using a scoring formula called H-Score.

H-Score = Total (Degree of staining + 1) × (Percentage of cells stained at each degree)

### 2.8. Histochemical Staining

Tibia bone tissues were fixed with 10% neutral formalin. Then, they were demineralized and washed under tap water. Sections with 5 µm thickness were cut from the paraffin blocks and stained with hematoxylin–eosin. The sections were kept in an oven overnight to remove paraffin and then in xylene for one hour. After being passed through a descending ethyl alcohol series (100–70%) and put into distilled water, the sections were kept in hematoxylin for 5 min. Later, the sections were washed in tap water for 10 min and stained with eosin for 2 min. Then, the sections were put into distilled water and passed through increasing alcohol series (100–70%) and xylene, respectively. Histochemical evaluation of all sections was performed under a light microscope (Eclipse E-600 Nikon, Japan) and analyzed with an image system (NIS Elements Nikon, Japan). Osteoblast surface (Ob.S/BS, unit = %), osteoclast surface (Oc.S/BS, unit = %), eroded surface (ES/BS, unit = %), and osteoid surface (OS/BS, unit = %) were measured.

### 2.9. Statistical Analysis

Gene expressions of diabetes and diabetes + MF groups were normalized to the mean of the control groups. When the diabetes + MF group was compared to the diabetes group, related data were also normalized with the corresponding GAPDH. All variables were expressed as mean ± standard error of the mean (SEM). Statistical comparisons were performed using one-way ANOVA followed by an appropriate post hoc test (Tukey). Two-tailed *p* values less than 0.05 were accepted as statistically significant.

## 3. Results

### 3.1. Metabolic and Biochemical Parameters

Table 2 displays the metabolic and biochemical parameters of the rats in the control, diabetes, and diabetes + MF groups. All groups were statistically similar regarding initial weight but there was a statistically significant increase between the initial and final weight for all groups. The diabetes group had a significantly higher final weight than the control group and this increase was significantly reduced by MF administration. 

When compared to the healthy rats, serum concentrations of glucose, triglyceride, VLDL, ALT, ALP, creatinine, urea, uric acid, and phosphorus were elevated significantly in diabetic rats. This elevation was significantly normalized with MF supplementation. Serum Na levels were significantly lower in the diabetic rats than the healthy rats, but this decrease was significantly reversed with MF treatment. 

### 3.2. mRNA Expressions of Bone Markers

**Figure 2:**
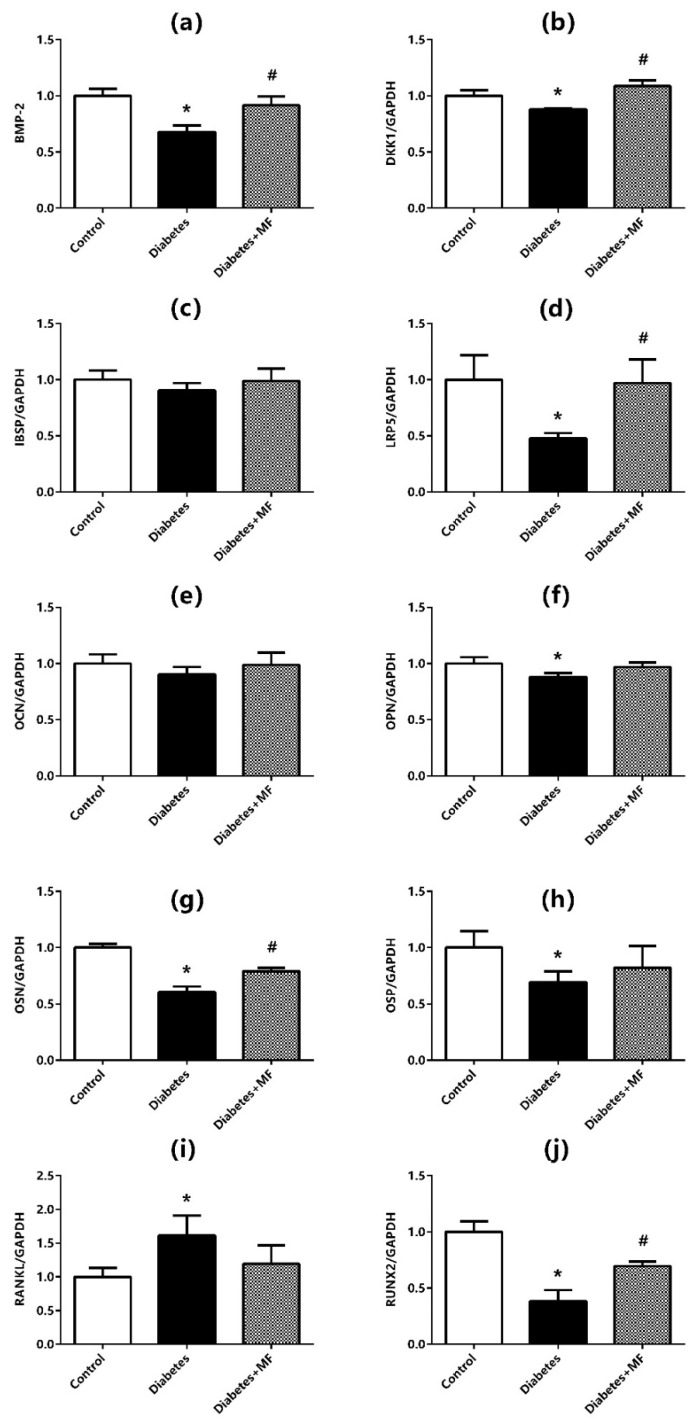
mRNA expressions of BMP-2 (**a**), DKK1 (**b**), IBSP (**c**), LRP5 (**d**), OCN (**e**), OPN (**f**), OSN (**g**), OSP (**h**), RANKL (**i**), and RUNX2 (**j**) at the bone–implant site of rats in the control, diabetes, and diabetes + MF groups. Data were normalized by GAPDH. Each bar represents the means of at least six rats. Values are expressed as mean ± SEM, *n* = 6–8. * Significantly different (*p* < 0.05) compared to control group; # significantly different (*p* < 0.05) compared to diabetes group.

The mRNA expressions of BMP-2 (Figure 2a), DKK1 (Figure 2b), IBSP (Figure 2c), LRP5 (Figure 2d), OCN (Figure 2e), OPN (Figure 2f), OSN (Figure 2g), OSP (Figure 2h), RANKL (Figure 2i), and RUNX2 (Figure 2j) at the bone–implant site were measured by real-time PCR. The mRNA expressions of BMP-2, DKK1, LRP5, OPN, OSN, OSP, and RUNX2 were significantly decreased, and mRNA expression of RANKL was significantly increased in diabetic rats compared to healthy rats. However, there was a significant increase in mRNA expressions of BMP-2, DKK1, LRP5, and OSN in MF-fed diabetic rats compared to diabetic rats. Although mRNA expressions of OPN and OSP tended to increase and mRNA expression of RANKL tended to decrease in diabetic rats fed with MF, these alterations were not statistically significant. No change was observed in mRNA expressions of IBSP and OCN at bone–implant sites of the rats in control, diabetes, and diabetes + MF groups. 

### 3.3. Imaging of the Bone–Implant Complex

**Figure 3:**
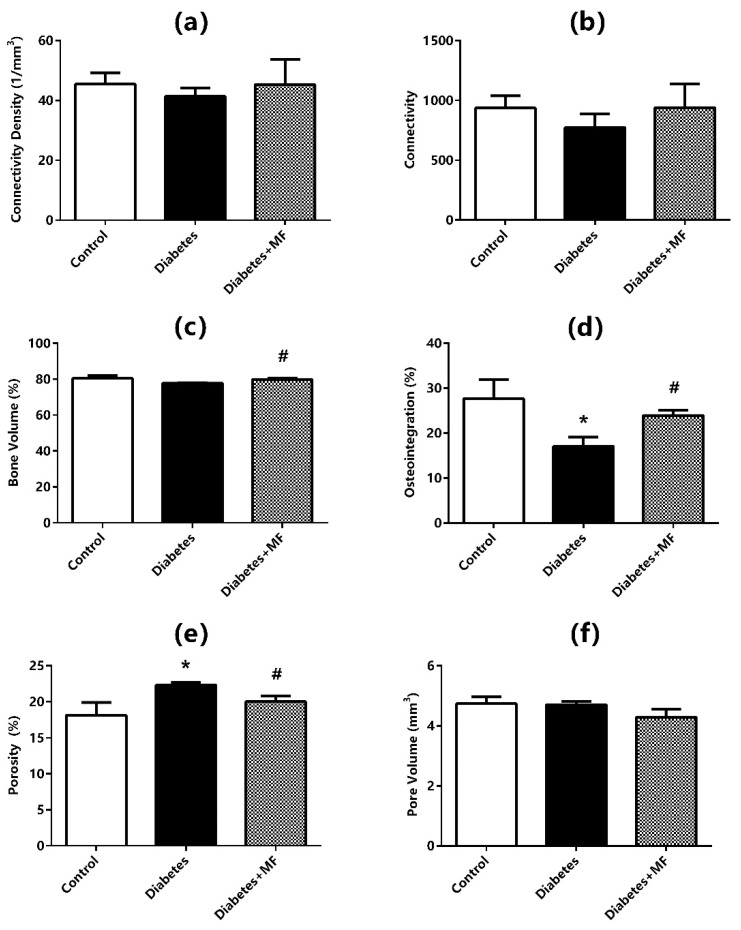
Imaging of connectivity densities (**a**), connectivity (**b**), bone volume (**c**), osteointegration percentage (**d**), porosity (**e**), and the pore volume (**f**) at the bone–implant site of rats in the control, diabetes, and diabetes + MF groups. Each bar represents the means of at least six rats. Values are expressed as mean ± SEM, *n* = 6–8. * Significantly different (*p* < 0.05) compared to control group; # significantly different (*p* < 0.05) compared to diabetes group.

**Figure 4:**
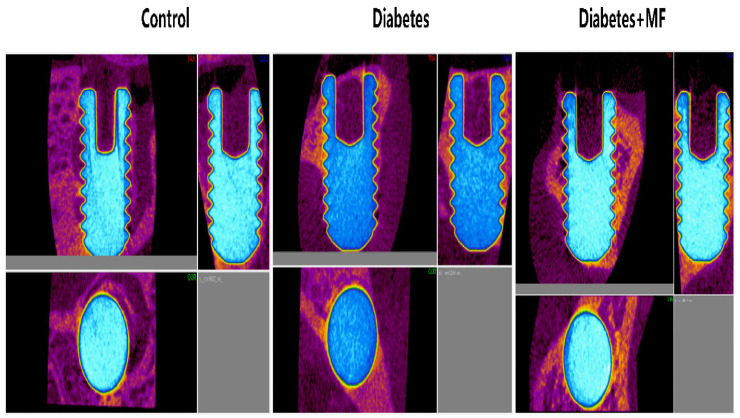
Representative imaging of the tibia bone–implant complex in the control, diabetes, and diabetes + MF groups.

Connectivity density (Figure 3a), connectivity (Figure 3b), bone volume (Figure 3c), osteointegration percentage (Figure 3d), porosity (Figure 3e), and total pore volumes (Figure 3f) are presented with radiological imaging of the bone–implant complex in all groups (Figure 4). The control, diabetes, and diabetes + MF groups were statistically similar with respect to the connectivity density, connectivity, and bone volume measurements of the bone–implant complex. However, the connectivity density, connectivity, and bone volume tended to decrease in diabetic rats compared to healthy rats and the relative decrease in these three radiological parameters of diabetic rats was compensated by MF supplementation. The osteointegration percentages of the bone–implant complex were significantly reduced in diabetic rats compared to healthy rats, but the significant decrease in osteointegration percentages was significantly reversed in diabetic rats fed with MF. Although the porosity percentages of the bone–implant complex were elevated significantly in diabetic rats compared to healthy rats, MF administration significantly normalized this elevation in diabetic rats. The control, diabetes, and diabetes + MF groups were statistically similar regarding total pore volumes. 

### 3.4. Immunohistochemistry

**Figure 5:**
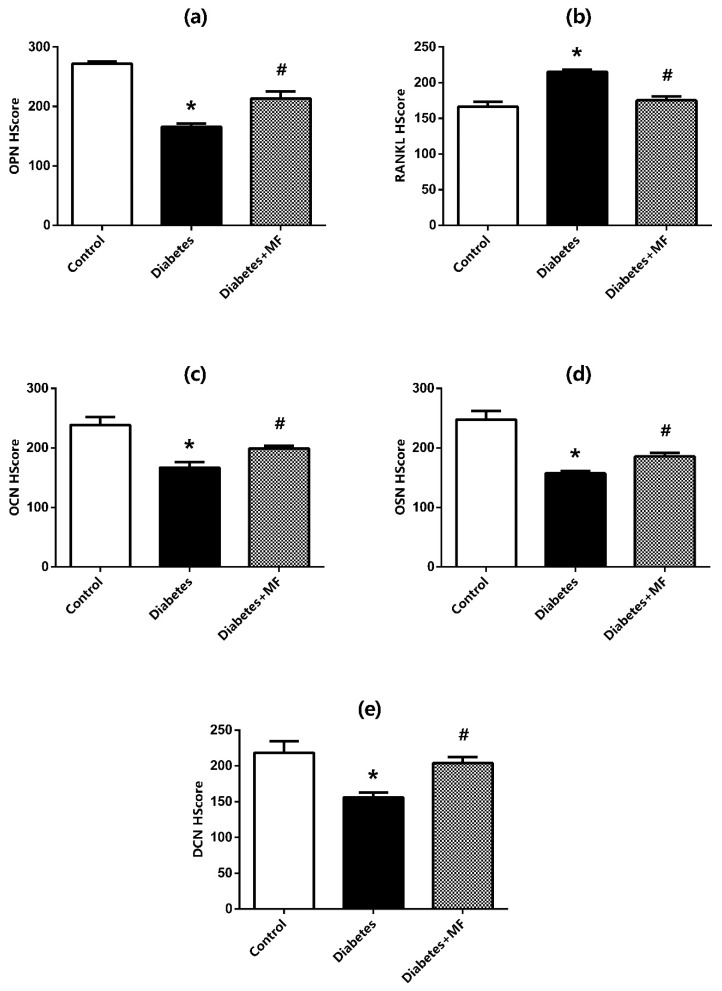
H-Score values for immunostaining by OPN (**a**), RANKL (**b**), OCN (**c**), OSN (**d**), and DCN (**e**) proteins at the bone–implant site of rats in the control, diabetes, and diabetes + MF groups. Each bar represents the means of at least six rats. Values are expressed as mean ± SEM, *n* = 6–8. * Significantly different (*p* < 0.05) compared to control group; # significantly different (*p* < 0.05) compared to diabetes group.

**Figure 6:**
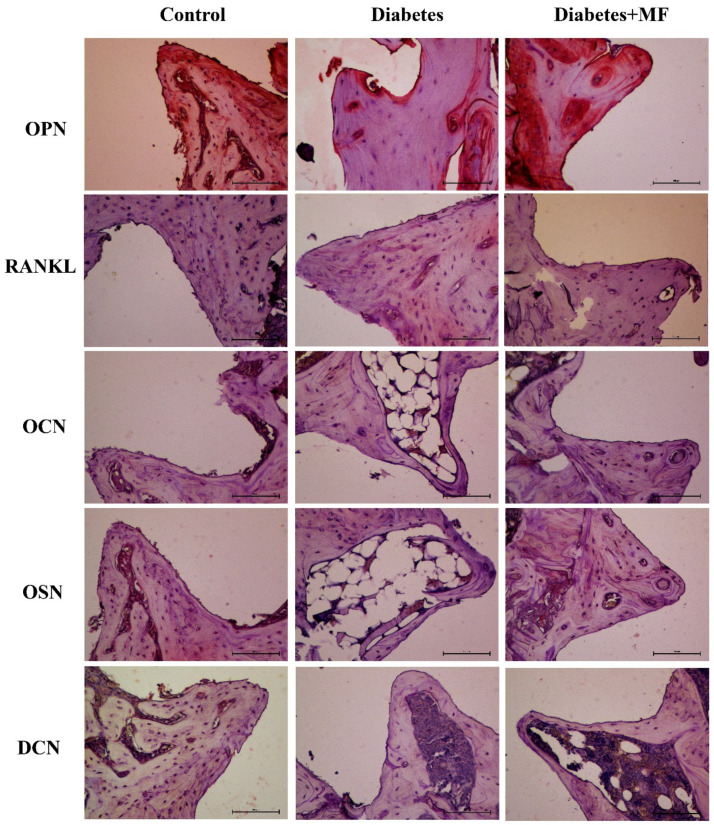
Immunostaining by OPN, RANKL, OCN, OSN, and DCN proteins at the bone–implant site of rats in the control, diabetes, and diabetes + MF groups.

H-Score values about the protein expressions of OPN (Figure 5a), RANKL (Figure 5b), OCN (Figure 5c), OSN (Figure 5d), and DCN (Figure 5e) at bone–implant sites of the rats are presented together with the related hematoxylin–eosin staining images (Figure 6). The expressions of OPN, OCN, OSN, and DCN decreased significantly in diabetic rats compared to healthy rats, and MF supplementation significantly reversed this decrease. The expression of RANKL increased significantly in diabetic rats and decreased significantly with MF treatment in these rats.

### 3.5. Histochemical Staining

**Figure 7:**
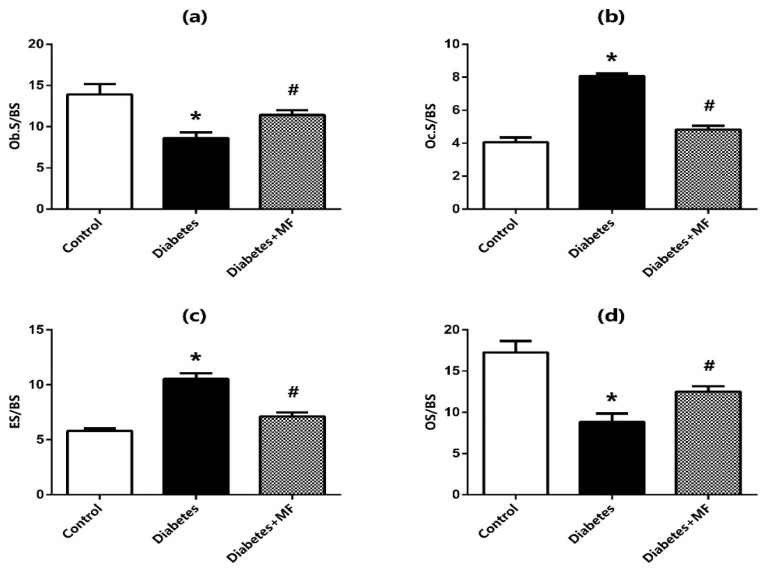
Histochemical staining of osteoblast surface (Ob.S/BS) (**a**), osteoclast surface (Oc.S/BS) (**b**), eroded surface (ES/BS) (**c**), and osteoid surface (OS/BS) (**d**) at the bone–implant site in the control, diabetes, and diabetes + MF groups. Values are expressed as mean ± SEM, *n* = 6–12. * Significantly different (*p* < 0.05) compared to control group; # significantly different (*p* < 0.05) compared to diabetes group.

**Figure 8:**
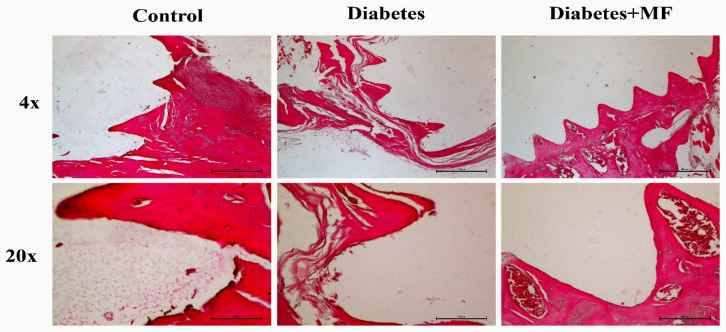
Histochemical staining in the control, diabetes, and diabetes + MF groups.

Histochemical stainings of osteoblast surface (Ob.S/BS) (Figure 7a), osteoclast surface (Oc.S/BS) (Figure 7b), eroded surface (ES/BS) (Figure 7c), and osteoid surface (OS/BS) (Figure 7d) at the bone–implant site of the rats in the control, diabetes, and diabetes + MF groups are presented together with hematoxylin–eosin staining images (Figure 8). The Ob.S/BS and OS/BS ratios were significantly decreased in diabetic rats compared to healthy controls and this decrease was significantly recovered by MF supplementation. The Oc.S/BS and ES/BS ratios were significantly elevated in diabetic rats compared to healthy rats and this elevation was significantly normalized by MF administration.

## 4. Discussion

Diabetes mellitus has a complicated etiopathogenesis which comprises genetic factors, organ failure, nutrition, physical activity, drug use, and hereditary predisposition [33]. However, the factors involved in both the pathogenesis of the disease and its complications open the door to many other pathologies including cardiovascular diseases [1]. Therefore, it is of great importance to provide symptomatic treatment of the disease and control disease-related factors, and especially hyperglycemia. Dysregulated blood glucose creates oxidative stress by causing the formation of advanced glycation products, moreover, it also affects lipid metabolism in the process by inducing glycogenesis [15]. Hyperglycemia causes acute symptoms such as polyuria, polydipsia, polyphagia or loss of appetite, weakness, rapid fatigue, dry mouth, nocturia, and impairment in wound healing. When hyperglycemia becomes chronic, cardiovascular diseases such as hypertension and endocrine disorders such as dyslipidemia might emerge [1]. 

The prolongation of the tissue regeneration process, especially in the post-operative period of hyperglycemic cases, makes clinicians anxious and reduces the treatment success rate. Dental implant applications are a long and laborious process for both the patient and the clinician [34]. The possible hazards of hyperglycemia in wound healing and bone regeneration in diabetic patients make the regulation of blood glucose even more important throughout recovery. In addition to oral hypoglycemic drugs, herbal extracts or bioactive compounds are the subject of research for diabetes treatment as it is usually considered that these extracts or compounds have a lower risk of side effects [35]. Thus, this study has been designed to investigate whether MF administration would have beneficial effects on the biochemical and metabolic parameters, mRNA expressions of bone markers, immunohistochemistry, and histochemical staining of bone tissue in a rat model of diabetes mellitus.

Blood glucose level and HbA1c value are amongst the most frequently adopted biochemical criteria for diabetes mellitus. As for the streptozocin-induced diabetes models in rodents, it is preferred that 50 mg/kg i.p. is usually administered for the type-I diabetes model [36], 35 mg/kg i.p. for type-II diabetes [37]. In this study, streptozotocin was given to the rats at a dose of 35 mg/kg and the existence of hyperglycemia was verified by the measurement of glucose in blood samples obtained from tail veins. Moreover, hyperglycemia was normalized by MF treatment in diabetic rats. This finding complied with a similar study conducted on mice, which indicated that MF reduced the high-fat-diet-induced blood glucose levels. [38]. 

Serum triglyceride, VLDL, ALT, AST, ALP, creatinine, urea, uric acid, and phosphorus levels are amongst the other biochemical parameters which are associated with diabetes mellitus [39]. In this study, serum triglyceride, VLDL, ALT, AST, ALP, creatinine, urea, uric acid, and phosphorus levels were found to be elevated significantly in intracardiac blood samples of the sacrificed diabetic rats. This finding confirms that the diabetes model was successfully maintained during the study period. It has been reported that serum Na decreases with diabetes, resulting in polyuria [39]. Accordingly, serum Na levels of diabetic rats tended to decrease in this study, but this change was not statistically significant. This discrepancy might be due to the variation in the amount of drinking water consumed. This study also found that serum triglyceride, VLDL, ALT, AST, ALP, creatinine, urea, uric acid, and phosphorus levels decreased significantly when diabetic rats were fed with MF. Similarly, a prior study demonstrated that these diabetes-related parameters are improved with MF treatment [40]. Therefore, it would be prudent to assume that MF supplementation might inhibit hyperglycemic tendency.

Providing optimal success in the application of dental implants depends on the osteointegration process. This process refers to the incorporation of the implant into the bone tissue and the subsequent bone healing process which occurs in response to the wound or damage in the bone tissue [41]. In this context, Wnt pathway components, which participate in bone regeneration, give clues about how healthy the process is. It has been declared that hyperglycemia reduces the mRNA expressions of IBSP, LRP5, OCN OPN, OSN, OSP, and RUNX2, increases the mRNA expression of RANKL, and does not change the expression of BMP-2, BMP-7, and DKK1 level in craniomandibular bone samples of young rats feeding on a high-fructose corn syrup diet [11]. Similarly, relative bone volume, trabecular thickness, trabecular separation, trabecular number, and, expectedly, bone–implant contact decreased significantly in a rat model of implant osteointegration which was further complicated by type-II diabetes mellitus [42]. Another study assessed the osteointegration of implants and claimed that bone volume, average trabecular number, and osteointegration percentage decreased significantly with type-II diabetes induced by streptozocin [43]. 

The present study measured mRNA expressions of BMP-2, DKK1, IBSP, LRP5, OCN, OPN, OSN, OSP, RANKL, and RUNX2 by a real-time PCR method, and osteointegration percentage, bone volume, total pore volume, porosity, relative connectivity, and connectivity density were also quantified. mRNA expressions of DKK1, BMP-2, OPN, OSN, RUNX2, LRP5, and OSP mRNA were decreased significantly, mRNA expression of RANKL was increased significantly, and mRNA expression of IBSP and OCN decreased relatively at the bone–implant site of diabetic rats. 

Additionally, the percentage of osteointegration was decreased significantly, bone volume, relative connectivity, and connectivity density were relatively reduced, and total pore volume was found unchanged in imaging studies of the diabetic rats. This finding indicates that the bone porosity of diabetic rats has increased significantly. Furthermore, real-time PCR findings and imaging results conform to each other and help to hypothesize that bone regeneration is suppressed in diabetic rats both at the molecular level and macroscopically in tissues. A body of evidence for this hypothesis is the immunohistochemistry findings of the present study. That is, H-Scores of OPN, OSN, and OCN were decreased and those of RANKL, which acts as the activator for osteoclasts, were increased significantly in diabetic rats. DCN is well known for its suppressive effect on tumorigenesis, angiogenesis, and growth factors. The decrease in H-Scores of DCN in this study point out the functional impairment in bone regeneration and wound healing of the diabetic rats. The aggravation in osteoclastic activity and functional impairment in bone regeneration can also be detected in the shrinkage of osteoblast and osteoid surfaces and simultaneous enlargement of osteoclast and eroded surfaces. Thus, it can be proposed that hyperglycemia appears to have a negative effect on the integration of implants into the bone tissue in diabetic rats. 

MF is believed to offer protection against hyperglycemia and subsequent development of diabetes mellitus through its antioxidant and anti-inflammatory effects [44]. It has been shown that these protective effects occur by activating the PI3K/AKT pathway [45,46]. On other hand, it has been attested that MF can prevent structural deformations in damaged tissues by inhibiting angiogenesis and tumorigenesis [46,47]. These inhibitory actions have been attributed to the inhibition of metalloproteinases [47] or the regulation of AMPK/NRLP3 [38] and SIRT1/Foxo3a [48] or the inhibition of Nf-κB and TNFα [49,50,51]. 

These properties suggest that MF might have a positive impact especially on wound healing [47,50]. In accordance, topically applied MF has been beneficial in diabetic ulcers [52]. Although these positive effects of MF on diabetes and wound healing are known, there is no study that examines the efficiency of MF on wound healing in the case that implant-related bone damage occurs in a diabetic individual. The present study highlights that diabetes decreases osteointegration percentage, reduces bone volume, and increases porosity whereas MF increases osteointegration percentage, enlarges bone volume, and normalizes porosity. On the other hand, mRNA expressions of DKK1, BMP-2, OSN, RUNX2, and LRP5, which decrease with diabetes in bone tissue, increase significantly with MF administration. This finding was also affirmed by immunohistochemical studies. In addition, osteoblast and osteoid surfaces, which shrink with diabetes, widen with MF treatment. Histochemical findings also reveal the rise in osteoclastic activity and the concurrent decline in porous areas. Current data suggest that the possible beneficial effects of MF on bone regeneration and osteointegration of diabetic rats may be mediated through the regulation of the *Wnt* signaling pathway in the liver [53,54].

The results of this study should be interpreted carefully as their power is limited by the relatively short experimentation time and absence of longitudinal data. The bioactivity of MF has been generally considered non-toxic because its reported oral LD50 value corresponds to 400 mg/kg in mice [17,18]. Nonetheless, there is a lack of data about the efficacy and safety of MF for clinical use in human beings. Another limitation is the scarcity of animal studies and clinical trials focusing on the use of MF in the treatment of diabetes mellitus and related complications.

## 5. Conclusions

This study claims that MF contributes to the osteointegration process of dental implant by alleviating the long-term hazards of hyperglycemia in bone tissue and exerting positive effects on bone regeneration in a rat model of streptozotocin-induced diabetes. This claim is based on the radiological findings, mRNA expressions achieved through real-time PCR, immunohistochemistry studies, and histochemical findings. Therefore, this claim could be the rationale behind the use of MF during the placement of dental implants in cases complicated by diabetes. Although the positive effects of MF on the osteointegration process are clearly seen, elucidating its mechanism of action in more detail through cell culture studies will enable bolder steps to be taken in addressing MF as a natural therapeutic agent and an alternative for daily clinical practice. Further large-scale research on more diverse populations has been warranted to clarify the effects of MF treatment on osteointegration and the regeneration of bone tissue.

## Figures and Tables

**Table 1:** Primer sequences of BMP-2, DKK1, IBSP, LRP5, OCN, OPN, OSN, OSP, RANKL, RUNX2, and the internal standard GAPDH used for specifying mRNA expression by qRT-PCR.

Gene	Forward Primer Sequence (5′ → 3′)	Reverse Primer Sequence (3′ → 5′)	Product Length (bp)
*BMP-2*	CACGAGAATGGACGTGCCC	GCTTCAGGCCAAACATGCTG	249
*DKK1*	CTCAGTGTGGCACTTACCTG	TCTGCACCCTAGAGACAAAAG	200
*IBSP*	AGGCAACGAGTACAACACTG	CATAGCCATGCCCCTTGTAG	116
*LRP5*	GAGATTGACTGCATCCCTGG	GAGACAGACAGCATCGCAG	159
*OCN*	CGCTACCTCAACAATGGACT	AACACATGCCCTAAACGGTG	166
*OPN*	CGATCGATAGTGCCGAGAAG	TGAAACTCGTGGCTCTGATG	112
*OSN*	GGAAGCTGCAGAAGAGATGG	GGTCTTGTTGTCATTGCTGC	286
*OSP*	CTAGACAAGCAGGGGTAGGT	AACGTTGGGGGCAATATCAA	222
*RANKL*	CCCATCGGGTTCCCATAAAG	CAGGTTATGCGAACTTGG GA	241
*RUNX2*	CGCCTCACAAACAACCACAG	TCACTGCACTGAAGAGGCTG	225
*GAPDH*	TGATGACATCAAGAAGGTGGTGAAG	TCCTTGGAGGCCATGTGGGCCAT	249

**Table 2:** Metabolic and biochemical parameters of the rats.

Groups	Control		Diabetes			Diabetes + MF		
	Initial Weight	Final Weight	Initial Weight	Final Weight	*p*	Initial Weight	Final Weight	*p*
Weight (g)	331 ± 6	400 ± 7	322 ± 5	447 ± 10 ^a^	0.003	328 ± 4	410 ± 9 ^b^	<0.0001
Glucose (mg/dL)	131.9 ± 6.8		156.1 ± 13.2		0.13	133.8 ± 6.4		0.16
Triglyceride (mg/dL)	67.4 ± 10.6		236.4 ± 2.5 ^a^		<0.0001	119.6 ± 3.8 ^b^		<0.0001
VLDL (mg/dL)	13.5 ± 2.1		47.3 ± 5 ^a^		<0.0001	23.8 ± 0.8 ^b^		0.001
ALT (U/L)	45 ± 6.2		64.1 ± 4.5 ^a^		0.03	45.1 ± 1.9 ^b^		0.003
AST (U/L)	106.3 ± 15		137.5 ± 18		0.21	116.6 ± 7.5 ^b^		0.001
ALP (U/L)	3.5 ± 0.2		10 ± 1.3 ^a^		0.0006	6.3 ± 0.2 ^b^		0.02
Creatinine (mg/dL)	0.34 ± 0.02		0.38 ± 0.02		0.19	0.32 ± 0.01 ^b^		0.02
Urea (mg/dL)	41.6 ± 1		50.5 ± 2.1 ^a^		0.003	37.8 ± 1.5 ^b^		0.0006
Uric acid (mg/dL)	0.61 ± 0.06		1.33 ± 0.05 ^a^		<0.0001	0.78 ± 0.09 ^b^		0.0003
Phosphorus (mg/dL)	3.2 ± 0.2		4.5 ± 0.3 ^a^		0.004	3.3 ± 0.05 ^b^		0.003
Na (mEq/L)	141.4 ± 0.5		137 ± 1.3 ^a^		0.01	141.1 ± 0.6 ^b^		0.01

^a^ Significantly different (*p* < 0.05) compared to control group; ^b^ significantly different (*p* < 0.05) compared to diabetes group.

## Data Availability

The data that support the findings of this study are available from the corresponding author upon reasonable request.

## Abbreviations

BMP 2–7, Bone morphogenetic protein 2–7; DKK, Dickkopf WNT signaling pathway inhibitor 1; GAPDH, Glyceraldehyde 3-phosphate dehydrogenase; HE, Hematoxylin–eosin; IBSP, Integrin-binding sialoprotein; LRP5, LDL receptor-related protein 5; Nf-κB, Nuclear factor kappa B; Ob.S/BS, Osteoblast surface; OS/BS, Osteoid surface; OCN, Osteocalcin; OPN, Osteopontin; OSN, Osteonectin; OSP, Osteoprotegerin; qRT-PCR, Quantitative real-time PCR; RANKL, Receptor activator of nuclear factor kappa Β ligand; RUNX2, Runt-related transcription factor 2; TNFα, Tumor necrosis factor-α.
